# Comparison of Verbal, Braille, and Tactile Methods in Overcoming Anxiety Before Dental Procedures in Visually Impaired Children: To Overcome Anxiety Using Cognition and Hands (TOUCH)

**DOI:** 10.7759/cureus.85810

**Published:** 2025-06-11

**Authors:** Sandhyarani Huddar, Uttkarsha Garad, Anil Patil, Sujatha P, Amit Kolekar, Mrunmayi Patil

**Affiliations:** 1 Pedodontics and Preventive Dentistry, Bharati Vidyapeeth (Deemed to be University) Dental College and Hospital, Sangli, IND

**Keywords:** braille, pit and fissure sealant, raghavendra madhuri sujata tactile scale, verbal-tactile, visually impaired children

## Abstract

Background: Dental anxiety assessment for visually impaired children is important. Having a clear understanding of a child's anxiety can help tailor the best approach to care for their individual needs.

Aim: This study, To Overcome anxiety Using Cognition & Hands (TOUCH), aimed to assess the anxiety levels of visually impaired children before and after administration of the sealant procedure, after explaining the pit and fissure sealant procedure through three different methods: verbal, verbal with Braille, and verbal with tactile demonstration.

Materials and methods: A total of 30 children aged 7-9 years were included in this study from a school for visually impaired children. Children were divided into three equal groups: (i) Group A - Verbal (control group), (ii) Group B - Verbal-Braille, and (iii) Group C - Verbal-Tactile. Pre- and post-intervention anxiety levels were measured in each group using the Raghavendra, Madhuri, Sujata Tactile Scale (RMS-TS) and pulse rate monitoring.

Results: All groups showed a decrease in anxiety, but a significant decrease in anxiety was found in the verbal-tactile group and verbal-braille group.

Conclusion: The study demonstrated that using a combination of verbal, braille, and tactile methods to explain the dental procedure effectively reduced anxiety in blind children.

## Introduction

Dental care is a vital component of overall health, but children with visual impairments often face unique challenges in accessing and understanding it. Due to their reliance on non-visual sensory input, these children may find it difficult to comprehend standard dental procedures, which can increase their anxiety and fear, particularly when the experience is unfamiliar [1]. Anxiety, in turn, can affect the child’s cooperation and potentially lead to negative dental experiences, which may discourage future visits and contribute to long-term oral health issues.

Among common pediatric dental procedures, pit and fissure sealant application is a fundamental preventive measure in the early management of dental caries [2]. Visually impaired children often experience increased pre-treatment anxiety due to the absence of visual cues and a limited understanding of dental procedures [3]. This lack of visual input can hinder their ability to fully grasp the steps involved, making it more challenging to prepare them adequately for treatment. Given that dental anxiety is a prevalent concern among children, it is important to explore alternative methods of communication that cater to the specific needs of visually impaired children.

Advancements in tactile learning methods, such as braille and model-based techniques, have shown promising results in enhancing understanding and reducing anxiety among children with visual impairments across various educational settings. Braille, a tactile writing system, allows visually impaired individuals to access written information independently, while tactile models offer a hands-on, interactive way to explain concepts or procedures by letting children feel and manipulate representations. These methods have proven effective in improving comprehension and reducing anxiety [4]. These approaches may not only help children better understand dental procedures but could also ease anxiety by offering more tangible and relatable explanations.

Despite the potential of these methods, limited research exists on their application within the context of dental procedures for visually impaired children. Hence, this study was planned to evaluate the effectiveness of three distinct instructional strategies for conveying information about a pit and fissure sealant procedure to visually impaired children: a traditional verbal explanation, a braille-based written explanation, and a tactile model-based demonstration. By comparing these methods, the study seeks to determine which approach is most effective in reducing anxiety and improving comprehension, thereby enhancing the dental experience for this vulnerable group.

The Raghavendra, Madhuri, Sujata Tactile Scale (RMS-TS) is a recently developed tool designed to assess anxiety in visually impaired children and consists of a series of five carved faces displaying expressions that range from not anxious to extremely anxious, scored from 1 (not anxious) to 5 (extremely anxious) [5]. It is named after its inventors, Raghavendra, Madhuri, and Sujata, and specifically created to address the needs of this population [1]. The RMS-TS (patented; Ref: No. 201741038533/CHE/2017) is a modified version of the RMS Pictorial Scale (RMS-PS) [6], utilizing tactile sensation instead of visual cues.

This study, To Overcome anxiety Using Cognition & Hands (TOUCH), is important because it provides valuable insights into how healthcare professionals can modify their communication strategies to better serve children with visual impairments, improving not only their dental care but also their overall health literacy and experiences in medical settings. The null hypothesis is that tailored communication strategies will have no significant impact on the dental experiences or health literacy of children with visual impairments.

## Materials and methods

This was a cross-sectional study, conducted at the Bharati Vidyapeeth (Deemed to be University) Dental College and Hospital, Sangli, Maharashtra, India, from April 3 to April 5, 2025. Visually impaired children, aged 7-9 years, were selected from a residential Blind School. Prior to the commencement of the study, ethical approval was obtained from the Institutional Ethical Committee of Bharati Vidyapeeth (Deemed to be University) (approval number: BV (DU)MC&H/Sangli/IEC/D-140). Informed consent was secured from the children's caretakers, and assent was obtained from the children after explaining the study procedures in an age-appropriate manner.

Eligibility criteria

Visually impaired children with no previous dental experience, aged 7-9 years, with caries-free permanent molars requiring pit and fissure sealants were included in the study, while children whose parents declined participation or who had a history of other systemic diseases were excluded from the study. 

Study participants

A sample size of 30 was estimated as per a previous study [1]. The 30 children were divided into three groups in alphabetical order of names by the principal investigator. Group A (Verbal Group) was given a traditional verbal explanation about the procedure, Group B (Verbal-Braille Group) was given a braille-based written explanation along with a verbal explanation, and Group C (Verbal-Tactile Group) was given a tactile model-based demonstration along with the verbal explanation.

Study procedure

**Figure 1:**
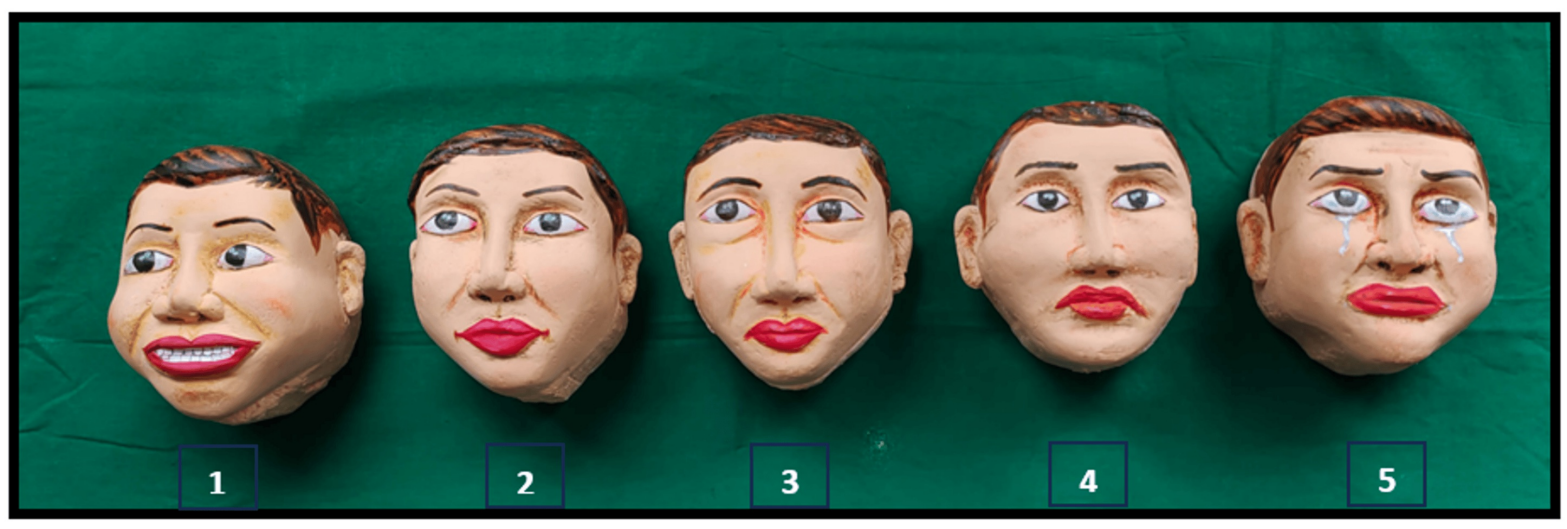
Raghavendra, Madhuri, Sujata Tactile Scale (RMS-TS) 1-not anxious, 2-slightly anxious, 3-fairly anxious, 4-very anxious, 5-extremely anxious

**Figure 2:**
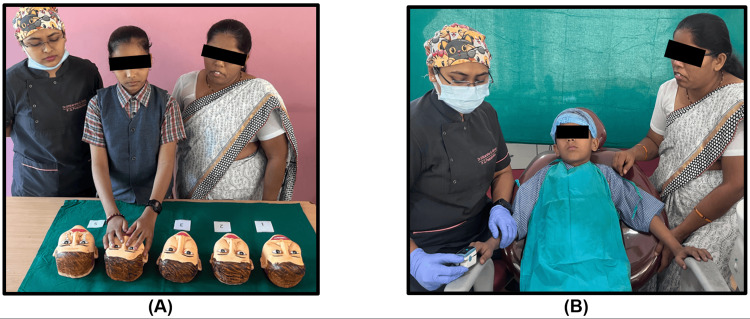
Anxiety assessment with (A) RMS-TS and (B) pulse rate measurement RMS-TS: Raghavendra, Madhuri, Sujata Tactile Scale [5]

Initially, the anxiety levels of the participants were assessed using the RMS-TS (Figures 1, 2A), and their pulse rates were measured (Figure 2B).

**Figure 3:**
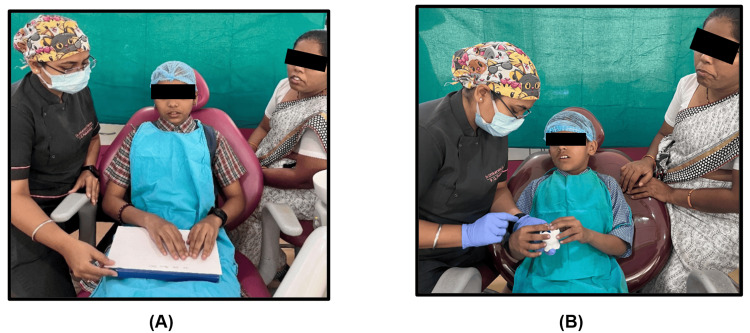
Along with verbal explanantions, (A) Group B participants were given a braille-based written explanation, and (B) Group C participants were given a tactile model-based demonstration

The participants were then given an explanation of the pit and fissure sealant (PF Seal; Prevest DenPro Limited, Jammu, Jammu & Kashmir, India) procedure (according to the manufacturer's instructions). Group A (Verbal Group) was a control Group, and the children received a verbal explanation of the pit and fissure sealant procedure. Children in Group B (Verbal-Braille Group) received verbal explanation of the pit and fissure sealant procedure, supplemented by a Braille script for enhanced comprehension (Figure 3A). Group C (Verbal-Tactile Group) received verbal explanation of the pit and fissure sealant procedure, along with the use of models to demonstrate the process with tactile sensation (Figure 3B).

**Figure 4:**
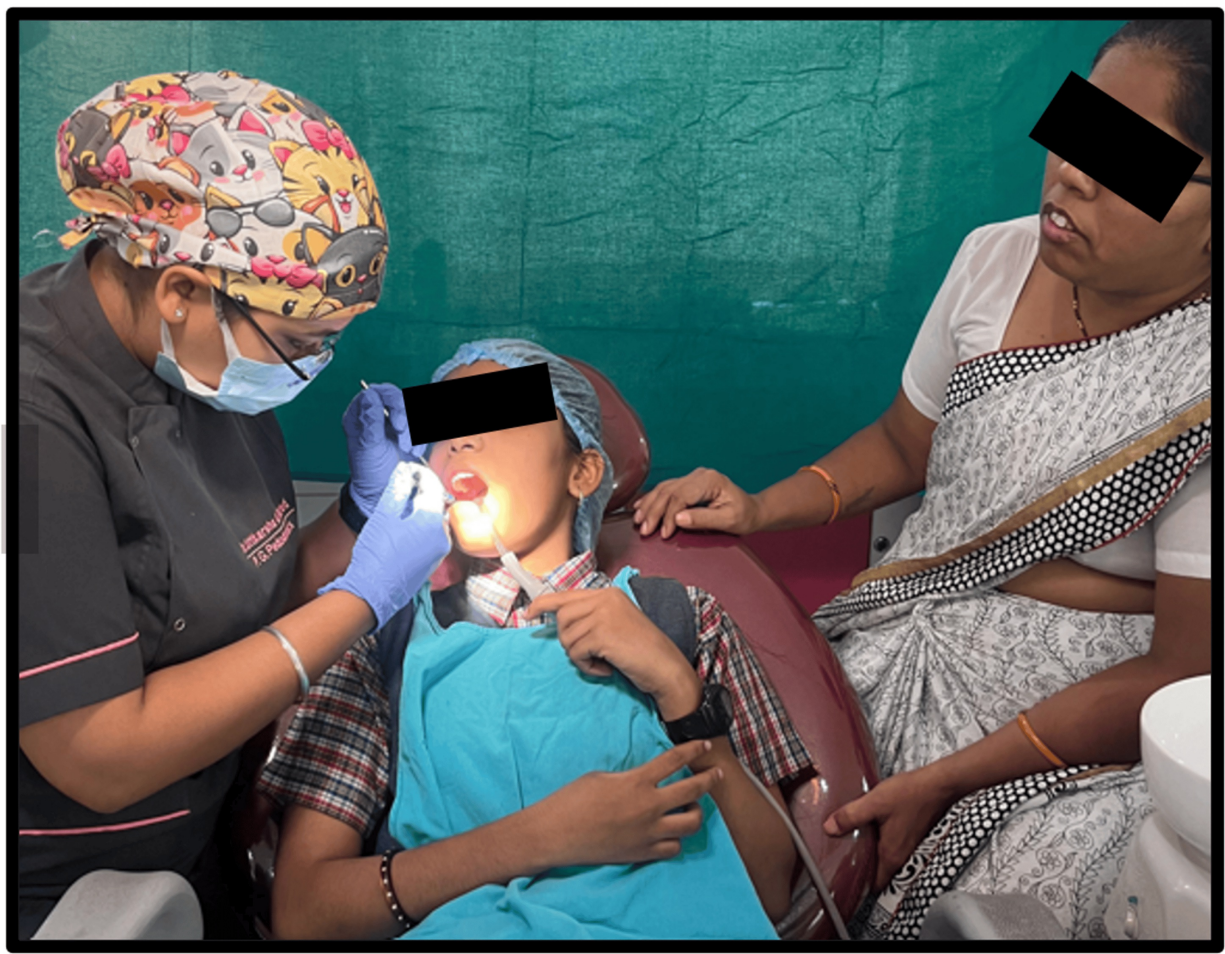
Pit and fissure sealant application

The sealant was then applied (Figure 4), and their anxiety levels were reassessed as shown in Figure 2.

Data analysis

Data was entered into a Microsoft Excel worksheet (Microsoft Corporation, Redmond, Washington, United States). Data analysis was done using IBM SPSS Statistics for Windows, Version 21.0 (Released 2012; IBM Corp., Armonk, New York, United States). Continuous data were presented as mean and standard deviation (SD). Intra-group comparison of means was done using a paired ‘t’ test, and inter-group comparison was done by one-way ANOVA. Data normality was checked by using the Shapiro-Wilk test. Overall intergroup comparison among the three groups was done using One-way Anova ‘F’ test followed by Tukey’s post hoc test for pairwise intergroup comparison between each group.

## Results

The effects of various interventions on RMS-TS scores along with inter-group and intra-group comparisons are shown in Tables 1, 2. For Group A (Verbal), the mean score reduced from 3.30 ± 0.82 before intervention to 2.30 ± 0.95 after intervention, with an intra-group p-value of 0.063, indicating no statistically significant change. In Group B (Verbal-Braille), the mean score significantly decreased from 3.00 ± 0.82 to 1.50 ± 0.53, with a p-value of <0.001. In Group C (Verbal-Tactile), the mean score significantly dropped from 3.40 ± 0.70 to 1.20 ± 0.42, with a p-value of <0.001. 

**Table 1:** Comparison of RMS-TS scores before and after intervention among the three groups (intra-group comparisons) p-value less than 0.05 considered statistically significant *statistically significant; **highly significant RMS-TS: Raghavendra, Madhuri, Sujata Tactile Scale

Group	Before intervention	After intervention	Intra-group comparison (p-value)
Group-A Verbal (Control), mean±SD	3.30±0.82	2.30±0.95	0.063
Group-B Verbal-Braille, mean±SD	3.00±0.82	1.50±0.53	<0.001**
Group-C Verbal-Tactile, mean±SD	3.40±0.70	1.20±0.42	<0.001**
F value	0.709	7.156	-
p value	0.501 NS	0.003*	-

**Table 2:** Inter-group comparison of RMS-TS scores before and after intervention among three groups p-value less than 0.05 considered statistically significant *Statistically significant RMS-TS: Raghavendra, Madhuri, Sujata Tactile Scale

Inter-group comparison	p value (Before intervention)			p value (After intervention)		
Group A vs Group B	0.671			0.034*		
Group A vs Group C	0.956			0.003*		
Group B vs Group C	0.496			0.584		

Before the intervention, the intergroup comparison revealed no statistically significant differences (F=0.709, p=0.501), with p-values of 0.671 for Group A vs. B, 0.956 for Group A vs. C, and 0.496 for Group B vs. C. After the intervention, the intergroup comparison revealed significant differences (F=7.156, p=0.003) between Group A and Group B (p=0.034) and Group A and Group C (p=0.003), while the comparison between Group B and Group C remained non-significant (p=0.584). 

Tables 3, 4 show the effects of various interventions on pulse rates along with inter-group and intra-group comparisons. In Group A (Verbal Control), the mean score decreased from 96.40 ± 5.21 before intervention to 89.20 ± 10.73 after intervention, with an intra-group p-value of 0.102, indicating no statistically significant change. In Group B (Verbal-Braille), the mean score significantly reduced from 95.30 ± 8.98 to 87.60 ± 9.06, with a p-value of <0.001. In Group C (Verbal-Tactile), the mean score significantly decreased from 97.90 ± 3.21 to 88.20 ± 4.57, with a p-value of <0.001. 

**Table 3:** Comparison of pulse rate before and after intervention among three groups (intra-group comparisons) p-value less than 0.05 considered statistically significant *Statistically significant; **Statistically highly significant

Group	Before intervention	After intervention	Intra group comparison (p-value)
Group-A Verbal (Control), mean±SD	96.40±5.21	89.20±10.73	0.102
Group-B Verbal -Braille, mean±SD	95.30±8.98	87.60±9.06	<0.001**
Group-C Verbal - Tactile, mean±SD	97.90±3.21	88.20±4.57	<0.001**
F value	0.432	0.09	-
p value	0.653	0.914	-

**Table 4:** Comparison of pulse rate before and after intervention among three techniques (inter-group comparisons) p-value less than 0.05 considered statistically significant

Inter-group comparison	p value (Before intervention)			p value (After intervention)		
Group A vs Group B	0.919			0.908		
Group A vs Group C	0.855			0.963		
Group B vs Group C	0.629			0.986		

Before the intervention, there were no statistically significant differences (F=0.432, p=0.653) between the groups (Group A vs. B: p=0.919, Group A vs. C: p=0.855, Group B vs. C: p=0.629). Similarly, after the intervention, no statistically significant differences (F=0.09, p=0.914) were observed (Group A vs. B: p=0.908, Group A vs. C: p=0.963, Group B vs. C: p=0.986). 

## Discussion

Pediatric dentistry is widely acknowledged as a specialized field that focuses on behaviour modification techniques, research, and development in providing dental care for children within dental settings. Unlike other areas, pediatric dentistry places particular emphasis on guidance and behavioural management. Treating children with medical fragility, such as those with visual impairments, presents unique challenges for pediatric dentists. Additionally, the anxiety levels in visually impaired children tend to be considerably higher compared to their sighted peers. It is crucial for pediatric dentists to understand the development of dental anxiety in children.

A study conducted by Pandey demonstrated that visually impaired students show significant differences in adjustment across home, school, and personal areas depending on their educational setting [7]. Students attending special schools and those in integrated schools exhibited varying levels of adaptation, highlighting the influence of the learning environment. These findings suggest that the type of educational setup plays a crucial role in the emotional and social adjustment of visually impaired adolescents. Therefore, tailored support strategies are essential to promote better adjustment outcomes among this population.

Nair et al. emphasized that vision plays a fundamental role in a child's development, and its absence can adversely affect overall growth, including oral health [8]. Although medical rehabilitation often takes precedence, access to dental care remains crucial for visually impaired children. With appropriate behavior management techniques, pediatric dentists can successfully provide comprehensive and quality dental care to this population.

In efforts to provide effective oral hygiene instructions for blind children, various methods such as braille, audio, and tactile techniques have been explored. Studies evaluating the effectiveness of these approaches in reducing anxiety have been conducted. A study by Hebbal et al. showed that using specialized techniques, such as audio-tactile methods, significantly helped reduce anxiety levels in visually impaired children [9]. Setiawati et al. conducted a study comparing the effectiveness of two dental health education methods, Braille Leaflet Dental Health Education (BLDHE) and Audio Dental Health Education (ADHE), for visually impaired children [4]. Their study concluded that both non-face-to-face educational techniques, BLDHE and ADHE, were effective in reducing dental anxiety in visually impaired children. Gautam et al. conducted a study to evaluate the impact of audio aids, braille, and tactile models on improving oral hygiene among visually impaired children [10]. Their study concluded that a combination of these methods was effective in delivering oral health education and improving the oral health status of visually impaired children. Krishnakumar et al. compared the effectiveness of audio and audio-tactile methods in improving the oral hygiene status of visually impaired schoolchildren [11]. The study found that when taught using customized techniques such as audio and audio-tactile methods, visually impaired children were able to maintain acceptable levels of oral hygiene.

Similarly, in the study by Mahantesha et al. to compare oral hygiene levels in institutionalized visually impaired children aged 6-20 years, they used braille and audio instructions, and their findings emphasized that continuous encouragement and reinforcement through Braille and audio-based education significantly contributed to maintaining good oral hygiene among visually impaired children [12]. Khan et al. conducted a study in which one group of visually impaired children received oral hygiene instructions in braille, while the other group received verbal instructions only [13]. Follow-up assessments at one and three months revealed that the group receiving braille instructions demonstrated a significant improvement in oral hygiene compared to the verbal-only group. These results suggest that Braille-based instruction is an effective method for improving oral health education in visually impaired children.

Rachman et al. proposed a three-pathway hypothesis to explain the development of fear, encompassing direct conditioning, modeling, and information processing [14]. Subsequent research, including studies by King et al. [15], has shown that these three pathways are interrelated; specifically, a strong correlation has been observed between dental fear and both the direct conditioning and modeling pathways. Dental phobia can complicate a child's treatment, making it more difficult for the dentist to proceed [16]. Thus, understanding anxiety levels in visually impaired children is essential, as it helps pediatric dentists employ the appropriate behaviour guidance techniques.

The neural pathways responsible for processing visual and tactile information are distinct. The primary visual cortex (V1) and the primary somatosensory cortex are separate regions in the cerebral cortex that independently process visual and tactile sensations. Higher-order cortical areas further integrate these sensory inputs, but still rely on the fundamental sensory cortices. Blindness results from the loss of V1, while tactile sensory abilities remain intact. Conversely, damage to the primary somatosensory cortex impairs the sense of touch but does not affect vision. Therefore, these two sensory pathways are distinct at the initial stages of cortical processing [17].

In this study, the RMS-TS method was used to assess anxiety along with pulse rate. Shetty et al. focused on validating the RMS-Tactile Scale (RMS-TS) as an anxiety assessment tool for visually impaired children by comparing it with a modified dental anxiety scale and a braille-based scale [5]. Using a set of five questions to assess emotions during various dental situations, they concluded that RMS-TS is a reliable tool for measuring dental anxiety in visually impaired children. Similar to our study, Kumbhar et al. evaluated the anxiety levels of visually impaired children using the RMS-TS after explaining the oral prophylaxis procedure either verbally or through a combined verbal-tactile method, followed by the actual procedure [1]. Their study concluded that the combination of verbal and tactile methods is more effective in reducing anxiety in visually impaired children.

No studies were found that specifically investigated the application of pit and fissure sealants for visually impaired children in a dental setting, while also recording anxiety levels during the procedure. Therefore, the focus of this study was to perform pit and fissure sealant applications on visually impaired children while recording their anxiety using the newer RMS-TS approach. The results of the present study indicated that children who received an explanation of the procedure using a combination of verbal-braille and verbal-tactile explanation methods exhibited a greater reduction in anxiety levels compared to those who were only provided a verbal explanation.

While the findings are encouraging, the small group of participants is a limitation. Future studies with more children could give us a clearer and more complete picture.

## Conclusions

Ensuring a positive and comfortable dental experience for individuals with disabilities is a critical component of equitable healthcare. To address dental anxiety and negative perceptions, it is essential to implement personalized, evidence-based educational strategies that accommodate each patient's unique sensory and communication needs. Everyone deserves the opportunity to care for their oral health; people with impairments should have the same support, understanding, and access as anyone else.

The present study shows that explaining dental procedures using a mix of verbal, braille, and tactile methods can really help ease anxiety in visually impaired children. Since this study was conducted with a small population, and given the positive results, there is further potential to conduct similar studies on a larger scale, as a smaller sample size limits generalizability. Future studies could focus on different dental treatments, such as restorations, extractions, and pulpectomies, as well as randomized control trials for visually impaired children. Utilizing the new RMS-TS aid, which incorporates various expressions to gauge the level of anxiety, would allow clinicians to better understand the child's anxiety and provide the best possible treatment in a calmer, more fearless, and less anxious manner.
